# NuMA Overexpression in Epithelial Ovarian Cancer

**DOI:** 10.1371/journal.pone.0038945

**Published:** 2012-06-14

**Authors:** Anke Brüning-Richardson, Jaqueline Bond, Rawiah Alsiary, Julie Richardson, David A. Cairns, Luci McCormac, Richard Hutson, Philip A. Burns, Nafisa Wilkinson, Geoff D. Hall, Ewan E. Morrison, Sandra M. Bell

**Affiliations:** 1 Section of Ophthalmology and Neuroscience, Leeds Institute of Molecular Medicine, St. James’s University Hospital, Leeds, United Kingdom; 2 Section of Oncology and Clinical Research, Leeds Institute of Molecular Medicine, St. James’s University Hospital, Leeds, United Kingdom; 3 Section of Pathology and Tumour Biology, Leeds Institute of Molecular Medicine, St. James’s University Hospital, Leeds, United Kingdom; 4 St James’s Institute of Oncology, Leeds, United Kingdom; University of Louisville, United States of America

## Abstract

Highly aneuploid tumours are common in epithelial ovarian cancers (EOC). We investigated whether NuMA expression was associated with this phenomenon.

NuMA protein levels in normal and tumour tissues, ovarian cell lines and primary cultures of malignant cells derived from ovarian ascitic fluids were analysed by Affymetrix microarray analysis, immunoblotting, immunohistochemistry (IHC) and immunofluorescence (IF), with results correlated to associated clinical data. Aneuploidy status in primary cultures was determined by FACS analysis.

Affymetrix microarray data indicated that NuMA was overexpressed in tumour tissue, primary cultures and cell lines compared to normal ovarian tissue. IHC revealed low to weak NuMA expression in normal tissues. Expression was upregulated in tumours, with a significant association with disease stage in mucinous EOC subtypes (p = 0.009), lymph node involvement (p = 0.03) and patient age (p = 0.04). Additional discontinuous data analysis revealed that high NuMA levels in tumours decreased with grade (p = 0.02) but increased with disease stage (p = 0.04) in serous EOC. NuMA expression decreased in late disease stage 4 endometrioid EOCs. High NuMA levels decreased with increased tumour invasion in all subtypes (p = 0.03). IF of primary cultures revealed that high NuMA levels at mitotic spindle poles were significantly associated with a decreased proportion of cells in cytokinesis (p = 0.05), increased binucleation (p = 0.021) and multinucleation (p = 0.007), and aneuploidy (p = 0.008).

NuMA is highly expressed in EOC tumours and high NuMA levels correlate with increases in mitotic defects and aneuploidy in primary cultures.

## Introduction

The 238 kDa nuclear mitotic apparatus (NuMA) protein is a component of the interphase nucleus and the mitotic spindle pole matrix [1]. NuMA has functions in spindle assembly through its association with microtubule motors [2], [3] and in spindle positioning during asymmetric cell division [4]. An additional interphase role as a nuclear scaffolding protein has been proposed [4]–[6]. NuMA is ubiquitously expressed [6], [7]. The nuclear and spindle pole localisations of NuMA are well characterised in normal tissue [8], [9] and in tumour tissues by immunohistochemistry (IHC) in the Human Protein Atlas. The role that mitotic proteins such as NuMA might play in cancer has recently become a subject of renewed interest as it has become apparent that aneuploidy is a common feature of tumours that might drive their progression [10], [11].

EOC is a complex disease that is difficult to treat due to late presentation, disease recurrence after treatment and high rates of chemo-resistance. The biology of EOC is poorly understood and improvements to diagnosis and therapy are highly desirable [12]. Ovarian carcinomas are often characterised by karyotypes with severe aneuploidy due to chromosomal instability (CIN) and triploidization [13]. It has been proposed that aneuploidy is a feature of early aberrations in EOC [14]. Aneuploid tumours respond less favourably to treatment than diploid tumours, leading to lower survival rates [15]. The *NUMA1* gene maps to chromosome 11q13 [16], a region commonly amplified in ovarian cancer [17]. However, to our knowledge no publications have linked NuMA expression to aneuploidy in EOC. We analysed NuMA levels in primary cultures of malignant cells derived from ovarian ascitic fluids, in their associated tumour tissues, in tissues from unrelated primary ovarian tumours and in normal ovarian tissue using Affymetrix array analysis, slot blotting, IHC and immunofluorescence (IF). NuMA expression was found to be upregulated in tumour samples compared to normal controls. IF analysis of primary cultures identified an association between increased amounts of NuMA at spindle poles and rates of binucleation and multinucleation, while FACS analysis revealed a positive correlation between increased NuMA expression and aneuploidy. This study demonstrates for the first time that NuMA expression is upregulated in EOC and that this is associated with the aneuploidy seen in this cancer.

## Results

### Affymetrix Microarray Analysis

**Figure 1:**
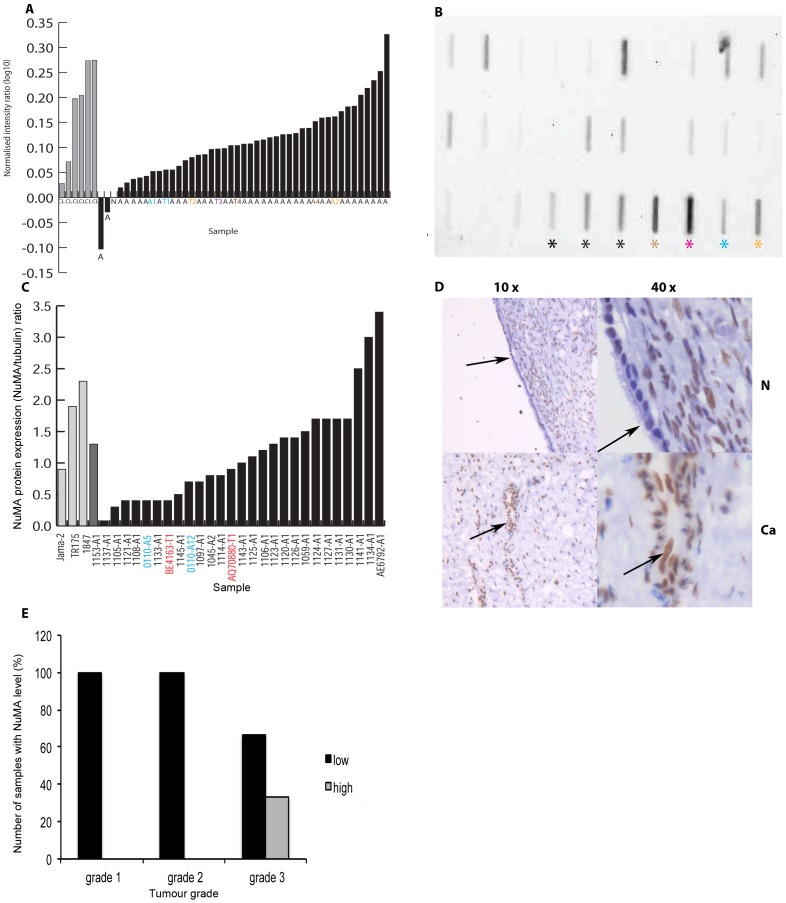
NuMA expression in ovarian cancer cell lines, primary cultures and tissue. A. Affymetrix data showing NuMA over-expression in 6 ovarian cell lines (JAMA-2, IOVE (additional immortalized ovarian epithelial cell line), SKOV-3, TR175, OVCA-433 (in addition) and 1847 (all grey bars, designated CL), 38 samples of primary cells cultured from ascitic fluid (black bars, designated – A, including some samples with consecutive collections or passages) and 4 primary cultures established from tumour tissue (designated –T1 to – T4). A1, A2 and A4 are tumour tissue (T1, T2, T4) associated ascites samples. Intensity values obtained for the samples were normalised to an internal control (normal epithelial ovarian tissue (N)) and gene expression levels are shown as the ratio of intensity levels/control intensity level as a log10 value. B. NuMA levels in cell lysates. A representative example of a lysates analysed for NuMA expression by slot blot Black asterix indicates ovarian cell lines (1847, TR175, JAMA-2), brown asterix the neuroblastoma cell line SH-SY5Y, pink asterix the colon adenocarcinoma cell line SW480, blue asterix the cervical cancer cell line HeLa, and yellow asterix the breast cancer cell line MCF7. All other samples are lysates from ovarian primary cultures. C. Histogram comparing NuMA levels in ovarian cell lines, a primary cell line derived from a benign gynaecological disorder (1153-A1) and primary cultures after normalisation against α-tubulin. D. Section of normal (N) ovarian epithelial and stromal tissue and matched ovarian cancer (Ca) at x10 and x40 magnification after NuMA immunostaining. Arrows indicate epithelial cells. Low to medium levels of nuclear NuMA staining can be seen in the normal epithelial cells, with higher levels of nuclear NuMA staining in the ovarian cancer epithelial cells. E. Analysis of a small ovarian TMA shows that NuMA staining intensity increases with grade in ovarian tumour tissue.

Examination of Affymetrix microarray data from a panel of ovarian cancer cell lines, primary cultures and tumour tissues showed that NuMA was consistently over-expressed at a transcriptional level in comparison with normal ovarian tissue. Only two ascitic samples had lower expression levels compared to an internal normal control (N) (Figure 1A). This initial work suggested that further study of NuMA expression in EOCs was warranted.

### Slot Blotting

A total of 35 samples were analysed by slot blotting. This cohort consisted of 7 established cell lines (JAMA-2, TR175, 1847, SW480, MCF-7, HeLa and SH-SY5Y), one benign sample (1153-A1) and 25 primary cultures. Lysates obtained from 2 ovarian tumour tissues grown in tissue culture (BE4163-T1 and AQ70880-T1) were also included. A representative example of a slot blot image of some of the samples is shown in Figure 1B. Normalisation against an internal α-tubulin control confirmed that there was variation in NuMA protein levels between the samples tested (Figure 1C). Among the cell lines, 1847 had the highest NuMA expression level, followed by TR175 and JAMA-2. The benign sample 1153-A1 had a medium NuMA level while there was variation amongst the primary ascites cultures with 3 (1141-A1, 1134-A1 and AE6792-A1) expressing the highest NuMA levels of all samples. No significant association between the NuMA immunoblotting results and the associated clinical data was seen. Reassuringly, NuMA protein levels in general corresponded well with the RNA expression levels seen for the primary cultures and ovarian cell lines examined by Affymetrix microarray analysis.

### Immunohistochemistry

**Figure 2:**
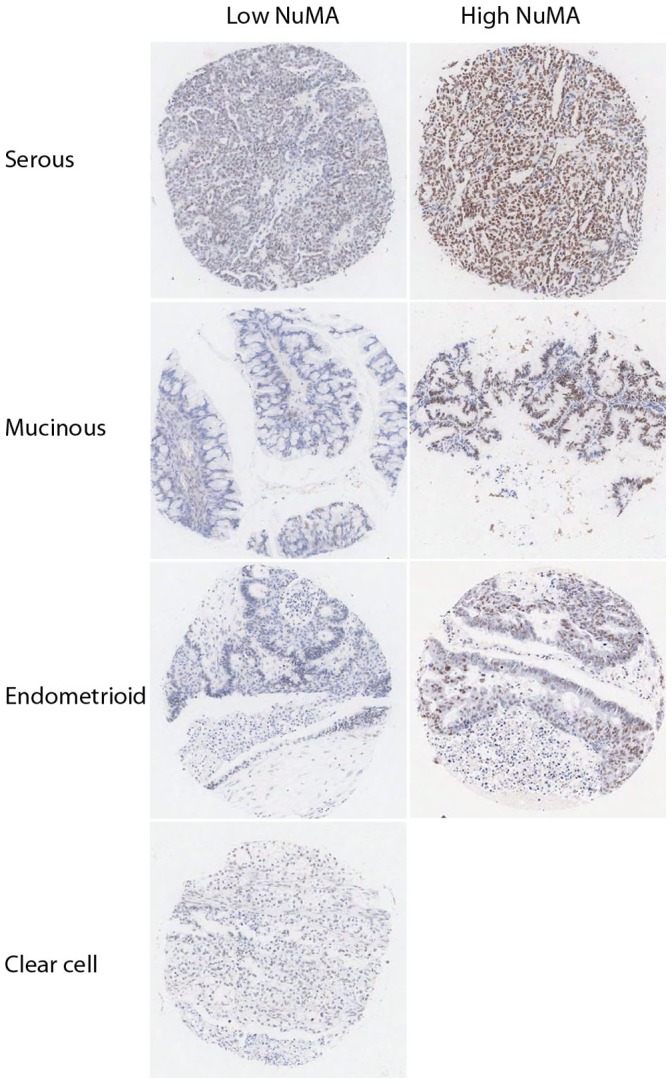
NuMA expression in different ovarian tumour subtypes. Low and high NuMA staining patterns observed in cores from serous, mucinous, endometrial and clear cell carcinomas in a large scale ovarian TMA. Magnification x10.

**Figure 3:**
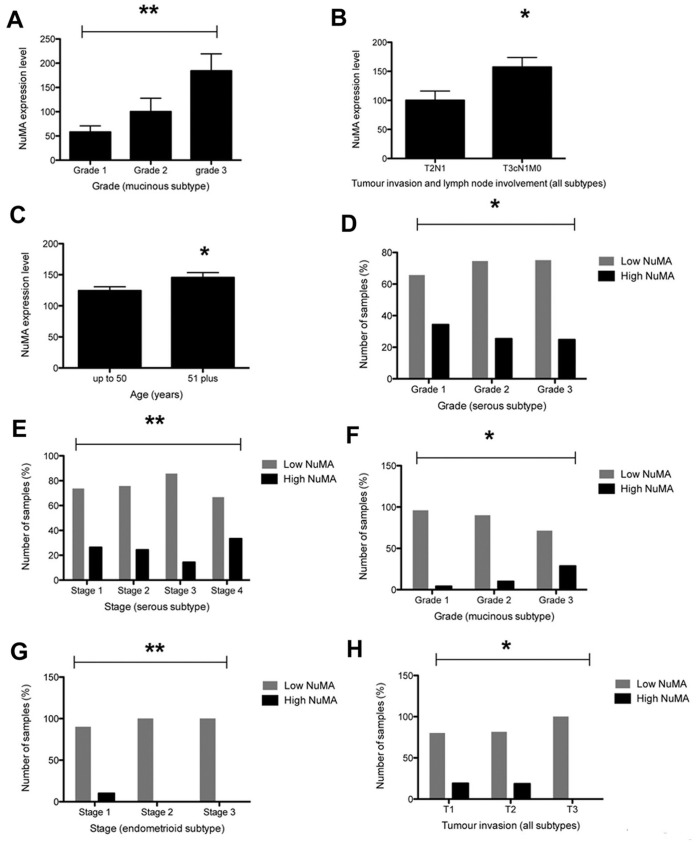
Analysis of NuMA expression in EOC. A. Statistical analysis of the large ovarian cancer TMA showing NuMA expression increases with disease stage in the mucinous subtype. B. NuMA expression is associated with lymph node involvement (T3cN1MO) (all subtypes). C. NuMA expression is associated with patient age (all subtypes). D. High levels of NuMA decrease with tumour grade in the serous subtype. E. High NuMA levels are associated with stage 4 disease in serous EOCs. F. High NuMA levels increase with tumour grade in mucinous EOCs. G. High NuMA levels decrease with disease stage in endometrioid EOC. H. High NuMA levels decrease with tumour invasion status (T1 to T3) (all subtypes).

Immunostaining indicated that NuMA expression in whole sections of normal ovarian tissue was variable, ranging from very low to weak levels in stromal and epithelial cell nuclei (n = 7) (Figure 1D). For the 5 cases of normal tissue with associated tumour tissue, a clear increase in NuMA expression in 40% (2/5) of tumour samples was identified. Up to 95% of nuclei in primary tumours displayed medium to strong NuMA staining (Figure S1a–d). NuMA was also observed at the spindle poles of mitotic tumour cells (Figure S1b, arrow). Analysis of a small in-house generated ovarian cancer TMA revealed that 95% of tumour cell nuclei were positively stained for NuMA in 24 of 25 cores. Strikingly, analysis of the data after application of a scoring system that subdivided samples into low NuMA (all negative, very weak and weak scores) and high NuMA levels (medium and high scores) showed that high NuMA levels were only detected in grade 3 tumours (33%; Figure 1E). Staining of a larger ovarian TMA confirmed low and high NuMA expression among the cores (Figure 2). Initial analysis of the overall data regardless of cancer subtype showed that high NuMA levels increased with tumour grade (from 20.3% for grade 1 tumours to 24.1% for grade 3 tumours, *p* = 0.0016) and disease stage (an increase from 19.6% for stage 1 to 33.3% for stage 4, *p* = 0.0077) (data not shown). Analysis of the continuous data for tumour grade and disease stage, which included subdivision into the cancer types for which enough data was available (serous, mucinous and endometrioid), indicated that NuMA expression correlated with increasing grade in the mucinous subtype (*p* = 0.009) (Figure 3A). A significant correlation with lymph node involvement (*p* = 0.03) (Figure 3B) and age (*p* = 0.04) (Figure 3C) was seen in all subtypes. Further analysis of the results as discontinuous data (low NuMA vs high NuMA) revealed additional statistically significant associations. The proportion of samples with high NuMA levels decreased with grade (*p* = 0.02) (Figure 3D) and increased in late disease stage in serous EOC (*p* = 0.04) (Figure 3E). In mucinous EOCs, NuMA levels increased with grade (*p* = 0.005) (Figure 3F). In late stage endometrioid EOCs, NuMA expression decreased (Figure 3G) and high NuMA decreased with an increase in tumour invasion status (*p* = 0.03) (all subtypes) (Figure 3H). Low NuMA expression was observed in the entire sample population of 13 clear cell EOCs regardless of disease stage.

### Immunofluorescence Analyses

**Figure 4:**
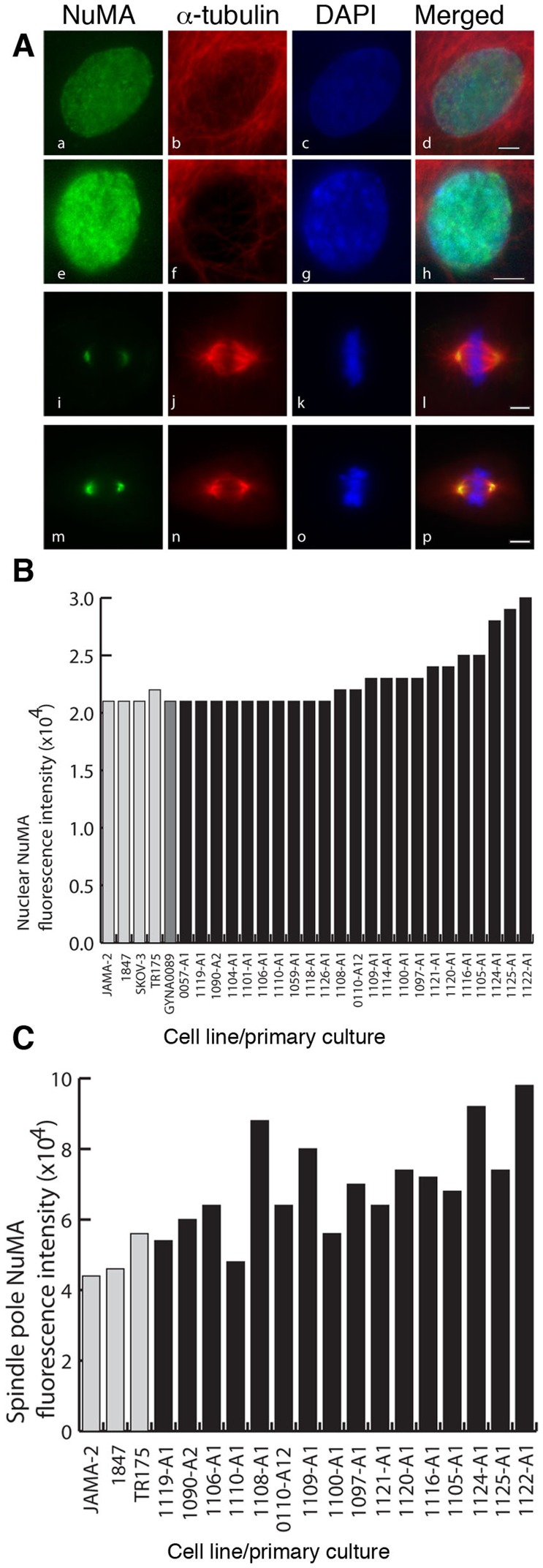
NuMA expression assessed by immunofluorescence reveals that NuMA localises to the nucleus during interphase and to the spindle poles during mitosis in cells of ovarian cancer cell lines and primary ascites cultures and fluorescence intensities vary among the primary cultures. A. Examples of interphase nuclei with low (a-d) and high (e-h) NuMA levels, and mitotic spindles with low (i-l) and high (m-p) NuMA levels at spindle poles. Green – NuMA, red – α-tubulin, blue - DAPI. Scale bar  = 5 µm. B. Histogram comparing NuMA nuclear fluorescence staining intensities in four ovarian cell lines, one cell line established from a primary culture (GYNA0089) and 23 primary cultures derived from ascitic fluid. C. Histogram comparing NuMA staining intensities at spindle poles in the subset of cell lines and cultures where a sufficient number of mitotic cells were for analysis.

IF analysis revealed the existence of an interphase nuclear NuMA staining pattern in all cell lines, one primary culture adapted as a cell line (GYNA0089) and 23 primary cultures (Figure 4A, B). In mitosis NuMA was found at the spindle poles (Figure 4C) in all of the cell lines and primary cultures examined. Sufficient numbers of mitotic cells for further analysis were identified in 16 primary cultures. In these samples dividing cells were examined for mitotic staging and the presence of specific mitotic defects. Finally, a survey of interphase indicators of aneuploidy was performed in all samples.

**Figure 5:**
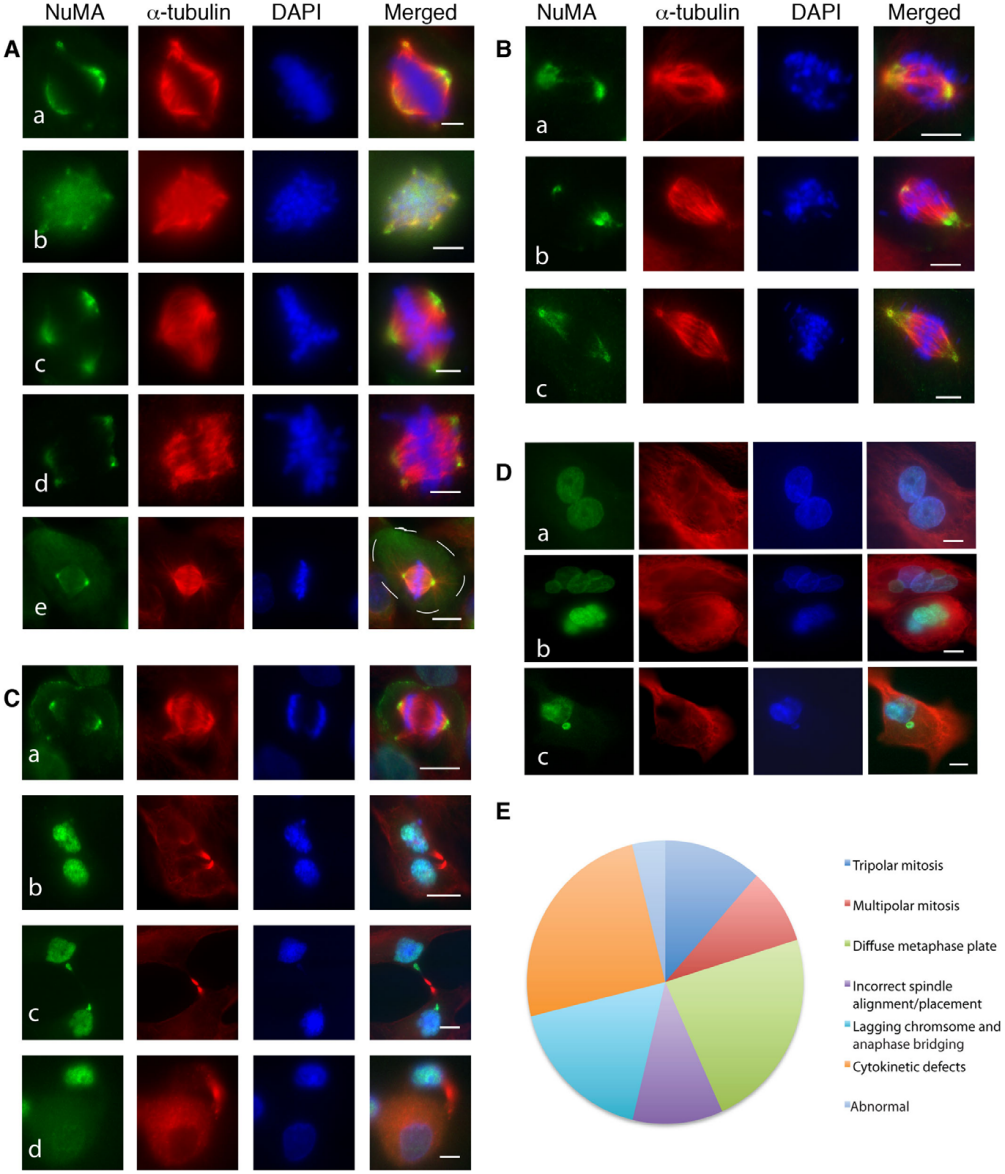
Mitotic and interphase defects in primary cultures. A. Mitotic defects observed at metaphase included (a) asymmetrical bipolar spindles (b) multiple spindle poles, (c) tripolar spindles, (d) bipolar spindles with multiple spindle poles (e) cells with misplaced and misaligned spindles (cell periphery outlined). B. Mitotic defects observed at anaphase included lagging chromosomes and anaphase bridging (a -c). C. Mitotic defects observed during telophase and cytokinesis included (a) abnormal spindles, (b) abnormal cytokinesis with micronuclei formation, (c), chromatid bridging and (d) formation of unevenly sized daughter cells. D. Examples of (a) binucleation, (b) multinucleation and (c) micronuclei in primary cultures. Green – NuMA, red – α-tubulin, blue – DAPI in all panels. Scale bar in A and B = 5 µm. Scale bar in C and D = 10 µm. E. Pie chart illustrating the overall frequency of mitotic defects observed in the primary cultures.

Staining intensities differed amongst the samples (Figure 4). It was noted that some samples were composed of cells with low level NuMA staining in interphase nuclei and at mitotic spindle poles (Figure 4A, a–d and i–l; 4B, C) and some samples were composed of cells with high NuMA staining intensities (Figure 4A, e–h and m–p; Figure 4B and C). We also noted that each sample differed with regards to the proportion of cells at each mitotic stage and that they exhibited a wide range of mitotic defects. These included metaphase defects such as tripolar and multipolar spindles (Figure 5A, a–d) and spindle misalignment (Figure 5A, e); anaphase defects such as lagging chromosomes and chromosomal bridging (Figure 5B, a–c); and cytokinetic defects including chromatid bridging and the generation of unevenly sized daughter cells (Figure 5C, a–d). Binucleation, multinucleation and the presence of micronuclei were common defects in interphase cells (Figure 5D, a–c). The examples shown in Figure 5E were the most common mitotic errors observed.

**Figure 6:**
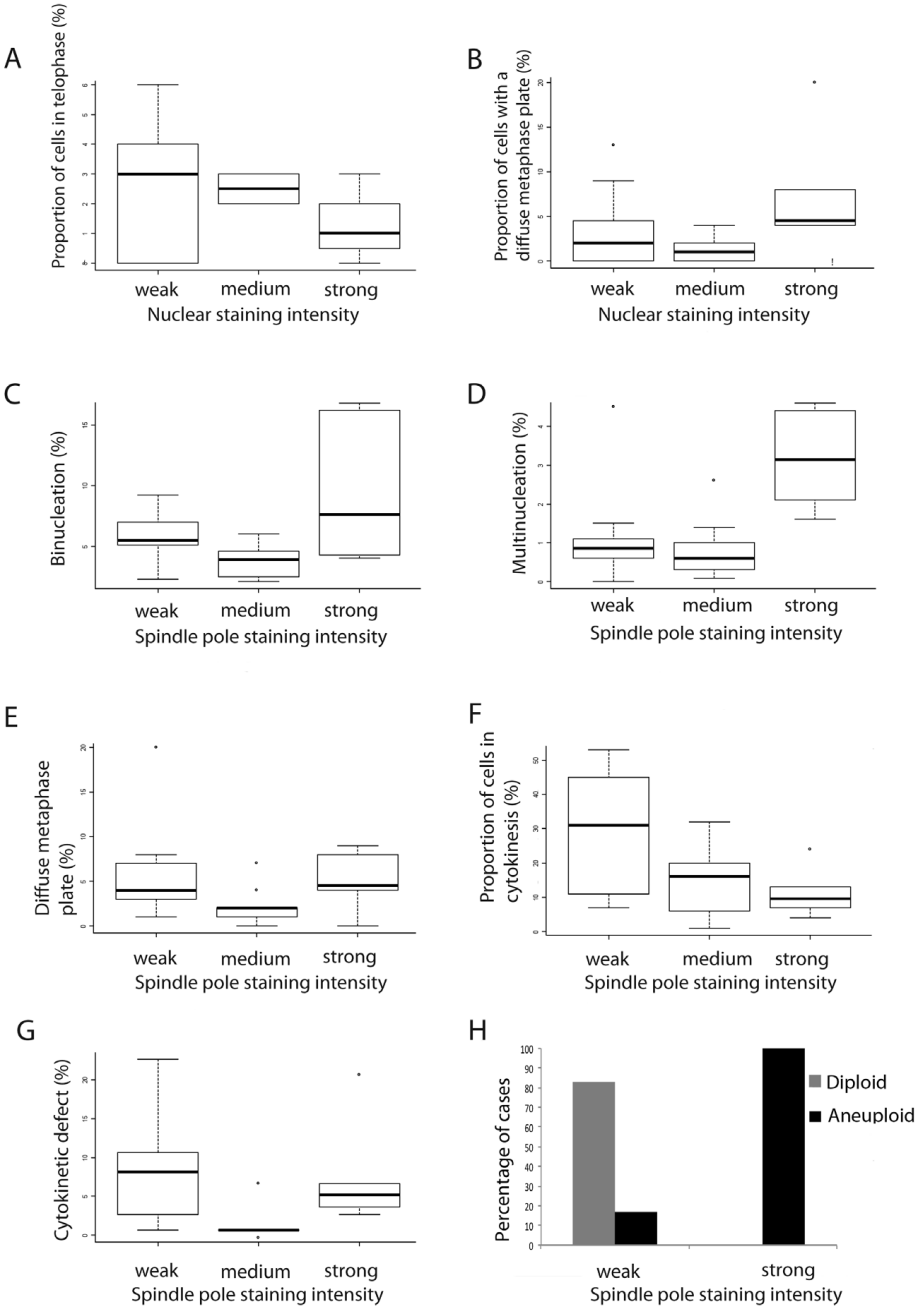
NuMA expression in primary cultures is associated with mitotic stage, mitotic defects and aneuploidy. Strong nuclear NuMA expression was associated with (A) the lowest proportion of telophase cells and (B) the highest proportion of cells with a diffuse metaphase plate. Strong NuMA spindle pole labelling was associated with high rates of (C) binucleation, (D) multinucleation, (E) a diffuse metaphase plate and (F) the lowest proportion of cells in cytokinesis. (G) Intermediate spindle pole staining was associated with the lowest proportion of cells exhibiting a cytokinesis defect. (H) FACS analysis revealed that only cells with high levels of NuMA were aneuploid.

A comparison of NuMA staining profiles to mitotic data was performed. High levels of NuMA in interphase nuclei were significantly associated with a small proportion of cells in telophase (*p* = 0.012) and a high proportion of cells displaying a loose metaphase plate (*p = *0.032) (Figure 6A and B). High levels of NuMA at mitotic spindle poles significantly correlated with high rates of binucleation (*p = *0.021) and multinucleation (*p = *0.007) (Figure 6C and D) and again with loose metaphase plates (*p = *0.041) (Figure 6E). Low levels of NuMA at spindle poles significantly correlated with a high proportion of cells in cytokinesis (*p = *0.05) (Figure 6F). Interestingly, intermediate levels of NuMA at the spindle poles significantly correlated with low error rates in cytokinesis. (*p = *0.01) (Figure 6G). To further verify the data observed in our immunofluorescence analysis of the primary cultures we immunostained whole sections of solid tumour tissue from the 23 patients whose ascitic fluids were used to generate the cultures. This confirmed that solid tumours displaying high levels of NuMA staining generated primary ascitic cultures where cells displayed high NuMA expression levels (Figure S1 a–d). To ascertain that the observed interphase and mitotic defects were also a common feature of the associated tumour tissues we analysed 23 tumour sections available to us by NuMA immunostaining and H&E IHC staining. We noted that the same interphase and mitotic defects were present in these tumours as in the ascites samples. Representative images and a summary of the defects observed among the samples are shown in Figure S2A–D. Furthermore, cell cultures with a high mitotic index were found to display high mitotic activity in the associated tumour tissue (Figure S3). Finally, we explored the relationship between NuMA expression levels in primary cultures and patient survival. This suggested that patients with moderate to strong NuMA immunostaining at the spindle poles in primary cultures have a lower survival rate than patients with weak NuMA immunostaining, though this preliminary data did not reach statistical significance (Figure S4), perhaps due to low sample size.

### FACS

FACS analysis indicated that only cells from primary cultures with high NuMA spindle pole staining intensities were aneuploid (Figure 6H), a statistically significant observation (*p = *0.008).

## Discussion

In this study we aimed to investigate *NuMA1* expression in EOC and relate this to aneuploidy found in this cancer type. An initial Oncomine search indicated that NuMA expression is upregulated in EOC. Our pilot data generated by Affymetrix profiling also suggested that *NuMA1* expression was upregulated in ovarian cancer. We confirmed this observation at the protein level by IHC, noting that nuclear NuMA was present at low levels in the nuclei of normal ovarian stroma and epithelium whereas ovarian primary tumours had elevated NuMA levels. High levels of NuMA were associated with tumour grade, disease stage, and lymph node involvement and inversely to tumour invasion. However, NuMA expression differed among the four major EOC subtypes. Whereas NuMA levels decreased with tumour grade and increased with disease stage in the serous subtype, they increased with tumour grade in the mucinous subtype and decreased with disease stage in the endometrioid subtype. In the clear cell subtype only low levels of NuMA were observed. These differences are likely to reflect the molecular heterogeneity of EOCs. Recently, EOCs have been reclassified according to their molecular features into type I (low grade serous, low grade endometrioid, all mucinous and clear cell carcinomas) and type II (high grade serous and endometrioid carcinomas) with mutations mainly in *KRAS* and *PTEN* in type I and mutations in *TP53* in type II [25], [26]. NuMA levels in our study were highest in type I EOCs (low grade serous and mucinous cancers), suggesting NuMA overexpression might be an early event in carcinogenesis.

Malignant cells derived from ascitic fluids have been used in the past to generate a wealth of information about immunology and therapeutics in EOC and it has been proposed that their use could complement conventional diagnostic procedures [27]. Here, the IF study of the primary cultures permitted a novel and detailed examination of evidence of aneuploidy through the identification of both specific mitotic defects and the interphase consequences of such defects. Interestingly, we found that high NuMA levels at spindle poles were associated with high levels of binucleation and multinucleation. The development of tetraploidy as a result of failed cytokinesis represents a potential early event in the development of chromosomal instability (CIN) and eventually aneuploid karyotypes in tumour cells [10]. The mitotic defects that we observed in our primary cultures, such as multipolar spindles and defects in cytokinesis, could explain the incidence of binucleated and multinucleated cells in these samples. Consistent with this, we also found a statistically significant correlation between aneuploidy and high levels of NuMA at the spindle poles in our cultures.

Having established that NuMA protein levels are upregulated in EOC and that this is associated with aneuploidy, the next step will be to determine the functional complicity of NuMA in the development of this aneuploidy. Work from other investigators has shown that conditional expression of NuMA in a mouse myeloid cell line results in aneuploidy and apoptosis [28]. It was suggested that NuMA overexpression alone was not sufficient to drive malignant transformation in leukemic cells but that it may contribute to chromosomal instability, allowing genetic events promoting cell cycle progression or protection from apoptosis to occur. One such event could be the acquisition of p53 mutations, such as those observed in high-grade serous carcinomas [29]. Alternatively, NuMA interacts with the motor protein dynein during mitosis and it has been suggested that saturation of dynein activity by NuMA overexpression might antagonise pathways that cancer cells use to resolve problems such as multipolar spindles. These typically arise due to the presence of supernumerary centrosomes, leading to an increase in genomic instability driven by defects in mitosis [30]. This hypothesis may be applicable to EOC since the biological consequences of multipolar cell divisions are consistent with the results observed in our immunofluorescence work. Centrosomal amplification has also been previously noted in EOC cells and has been linked to aneuploidy and poor patient outcome in serous EOC. This phenomenon appears to be caused by the overexpression of Aurora-A, a member of the serine/threonine kinase family involved in mitotic events [31], [32].

The tumour antigen acrosin binding protein (ACRBP)/OY-TES-1, has been shown to interact with NuMA. A co-dependent relationship with a role in paclitaxel resistance in EOC has been proposed [33]. NuMA overexpression was suggested to cause mitotic perturbations required for the plasticity of the preneoplastic genome, with co-evolving overexpression of ACRBP as tumours progress driving the acquisition of traits for the normalization of these early perturbations that could otherwise be disadvantageous for cancer cell proliferation by promoting mitotic catastrophe. The same study also revealed that most aggressive disease among a cohort of EOC patients was associated with combined high levels of ACRBP and NuMA. The possibility that NuMA overexpression acts synergistically with Aurora-A and ACRBP overexpression to promote aneuploidy, poor survival and chemoresistance in EOC is intriguing and worthy of further investigation. As part of this, our preliminary data suggesting that NuMA expression levels are associated with survival in EOC will be further explored in a larger sample set to determine whether this phenomenon is statistically significant.

In conclusion, our data demonstrates for the first time that NuMA is overexpressed in EOC and that high NuMA levels correlate with increased mitotic defects and aneuploidy in ascites cultures derived from patients with ovarian tumours.

## Materials and Methods

### Cell Lines and Primary Cultures of Malignant Cells Derived from Ovarian Ascitic Fluids

Cell lines used in this project included four ovarian cancer cell lines (JAMA-2, SKOV-3, TR175 and 1847), one neuroblastoma cell line (SH-SY5Y), one colon adenocarcinoma cell line (SW480), one cervical cancer cell line (HeLa) and one breast cancer cell line (MCF7). Cell lines were obtained from Cancer Research UK, the ECACC and the ATCC Cell Biology Collection. Primary cultures of cells derived from ovarian ascitic fluids (GYNA0089), ovarian tumour tissues (BE4163-T1 and AQ70880-T1) or benign tumours (1153-A1) were established as previously described [18]. Patient information and clinical data for the ovarian ascitic fluids has been previously described [18].

### Normal Ovarian and Tumour Tissue

Pathological specimens of formalin fixed, paraffin embedded tissues consisting of sections of normal ovarian tissue and primary tumours (including tumours that produced the ascites from which primary cultures were prepared) were obtained from St James’s University Hospital Histopathology Archive, Leeds. Seven normal tissues were available, 5 of which also had associated tumour samples. A total of 23 tumour tissues matching primary cultures derived from malignant cells were obtained. An additional 25 unrelated primary ovarian tumours with associated clinical data were identified and used to create an in-house mini tissue microarray (TMA). The TMA comprised of 9 serous adenocarcinomas, 6 endometrioid carcinomas, 2 mucinous, 2 mixed Mullerian, 5 mixed serous and endometrioid, and 1 adenocarcinoma. A high density ovarian cancer TMA consisting of 280 cases of ovarian adenocarcinoma (serous, mucinous and endometrioid), 13 clear cell carcinoma, one transitional cell carcinoma, 20 adjacent normal tissue and 8 normal tissues in duplicate, with stage and grade information, was obtained from Tissue Array Networks (OV6161, Tissue Array Network). Accompanying clinical data included patient age, lymph node involvement, tumour invasion and metastasis.

### Patient Data

Associated clinical details for patients for whom primary cultures of malignant cells derived from ovarian ascitic fluids were established, were described previously [18].

### Affymetrix Profiling

Data for ovarian cell lines, primary cultures and associated tumour samples was retrieved from a gene expression microarray analysis, generated with GeneChip HG-U133 Plus 2.0 Arrays (Affymetrix) that was normalised using MAS 5.0 as previously described [18].

### Antibodies

The NuMA monoclonal antibody Ab-2 (clone 107-7, Calbiochem) was used throughout this study. This has previously been extensively used for Western blotting, immunofluorescence and IHC [19], [20]. For IF studies rat anti-α-tubulin antibody (1/500, MCA77G, Serotec) was used to identify microtubules and 4′,6-diamidino-2-phenylindole (DAPI 5 µg/ml, Sigma-Aldrich) used to stain DNA.

### Slot Blotting

Slot blotting of cell lysates was performed as previously described [18]. 0.01 mg of sample was loaded in each well and the NuMA monoclonal antibody Ab-2 was used at a 1/400 dilution.

### TMA Construction

For the creation of an in-house ovarian TMA a protocol described by Abd El-Rehim *et al* [21] was followed. Manual tissue arrayer punches with a diameter of 0.6 mm (Beecher Instruments, Inc) were used to create the cores.

### Immunohistochemistry

Slides containing whole sections or TMA cores were blocked with 3% H_2_O_2_ in methanol after de-waxing and rehydration. Antigen retrieval was performed by pressure-cooking slides for 2 minutes in Antigen Unmasking Solution (low pH, Vector Laboratories). After three 5 minute washes in TBS-Tween-20 (0.2%) the slides were blocked for 10 minutes with 10× casein (Vector Laboratories) diluted 1∶2 in distilled water. After three 5 minute washes with TBS Tween-20 the slides containing whole sections were placed in Shandon sequenzer units whereas slides containing TMA sections were laid flat inside a humidified incubation chamber for incubation with the primary antibody. NuMA antibody Ab-2 diluted in antibody solution (Zymed) was applied at 1/300 for whole sections and 1/100 for the TMA slides. Slides were incubated overnight at 4°C. After three washes of 5 minutes each with TBS-Tween-20 the slides were incubated in 100 µl of HRP rabbit/mouse REAL™ EnVision™ (DAKO) for 30 minutes in the sequenzer units or incubation chamber. After three final washes with TBS for 5 minutes, developer solution (DAB+chromagen/substrate buffer, DAKO) was added for up to 10 minutes until colour development was complete. Staining was intensified in 0.5% CuSO4 (in 0.9% saline) for 5 minutes. After counterstaining in haematoxylin for 2 minutes, the sections were dehydrated and mounted in DPX (Sigma). TMA images were scanned and analyzed using Aperio ImageScope Viewer software. Slides containing whole sections were viewed with an Olympus BX61 microscope with Colourview camera and CellP® software. A labelling scoring system, which applied two scoring criteria, was utilised [22]. First, the number of stained nuclei were counted and expressed as a percentage of the total number of nuclei within the tissue section or core. Second, the predominant nuclear expression intensity was scored as 0 - no staining, 1 - trace, 2– weak, 3– medium and 4– strong. The final score was calculated as the percentage of stained nuclei multiplied by the predominant expression intensity. Expression intensity was correlated to associated clinical data including disease stage, tumour grade, cancer subtype, lymph node involvement, and metastatic status.

### Immunofluorescence

IF staining was performed as described previously [18]. The NuMA antibody was used at a 1/2000 dilution. To facilitate the identification and classification of mitotic and interphase defects, microtubules were visualised with a rat anti-α-tubulin antibody (1/500, MCA77G, Serotec) and DNA with 4′, 6-diamidino-2-phenylindole (DAPI 5 µg/ml).

### Indicators of Aneuploidy

Indicators of processes that might lead to aneuploidy, such as binucleation, multinucleation and the presence of micronuclei, were recorded by randomly scoring at least 600 DAPI-stained interphase cells from a minimum of four different fields of view for each IF labelled primary culture. The mitotic index for each culture was established by counting the total number of mitotic cells in each analysis and expressing this as a percentage of the total number of cells recorded. To determine the incidence, type [23] and stage (prophase, prometaphase, metaphase, anaphase, telophase and cytokinesis) of mitotic defects, a minimum of 50 mitotic cells were examined in each culture. Briefly, in prometaphase and metaphase any cell with tri- or multipolar spindles was recorded and metaphase cells with a loose metaphase plate and/or misplaced/misaligned spindles scored [24]. Cells with lagging chromosomes or chromosomal bridges were recorded as anaphase defects. Telophase and cytokinesis defects encompassed chromosomal bridging [23], the presence of abnormal midbodies and the observation of unequally sized daughter cells. All other mitotic defects were simply classified as “abnormal”.

### FACS Analysis of Aneuploidy

When cells reached confluence a single cell suspension was produced by trypsinization. The suspension was centrifuged at 1000 rpm for 5 minutes and supernatant discarded without disturbing the cell pellet. Cells were washed twice with PBS, centrifuged and the supernatant discarded. Cells were fixed by resuspension in 70% ice-cold ethanol for 30 minutes and were stored at −20°C until used in FACS analysis. After washing with PBS and resuspension in 900 µl PBS containing 100 µg/ml RNAse A, the cells were stained with 100 µl of 200 µg/ml propidium iodide (PI) and incubated for at least 30 minutes at 37°C. Cellular DNA content was measured on a BD LSRII analyser (Becton Dickinson). The DNA content of the samples was expressed as flow cytometric histograms using the ModFit LT software (Verity Software House). DNA aneuploidy was defined as any populations with distinct additional peaks (s) or when the DNA index (DI) was outside the normal diploid range of 0.9–1.1. The DI represents the ratio of the mean channel number of the tumour samples G0/G1 peak to the mean channel number of the normal lymphocyte control samples G0/G1 peak. Histograms with coefficients of variation greater than10% were excluded. At least 10,000 cells were counted in each sample.

### Statistical Analysis

For statistical analysis of the TMA data, Kruskall-Wallis and Mann-Whitney Tests (Prism 5 Graph Pad) were used to analyse the classifications of NuMA staining patterns in the TMA, ovarian cancer and normal ovarian tissue cores. A *p*-value of <0.05 was considered statistically significant. SPSS version 16.0 (SPSS Inc.) was used for comparison of the intensity of NuMA spindle pole staining and aneuploidy. The R environment for statistical computing (R Development Core Team) was used for some analysis of association between clinical variables and protein expression (Fisher’s Exact test and the Kruskall-Wallis test).

## Supporting Information

Figure S1 **NuMA staining patterns in primary tumours correspond to staining intensities observed in primary cultures.** Sections of primary tumour were immunostained for NuMA. (a,b) Weak nuclear staining was demonstrated in sample 1101; note spindle pole staining in mitotic cell (arrow) (b). (c,d) The strongest staining was observed in sample 1125. These findings mirror the results obtained by immunofluorescence analysis of the primary ascitic cultures derived from these tumours (Figure 4B).
(TIFF)

Figure S2 **Interphase and mitotic defects in cell cultures of malignant cells derived from ascitic fluids are also present in associated tumour tissues.** A. Binucleated and multinucleated cells and micronuclei are present in tumour tissues associated with ascitic cultures that also displayed these defects, as indicated by the NuMA and H&E staining. Number indicates patient ID, T indicates tumour sample. B. Various mitotic defects including multiple spindle poles, tripolar spindles, multipolar spindles and anaphase bridging are observed in cell cultures of malignant cells derived from ascitic fluids and in their associated tumours. IF images are from cell cultures of malignant cells derived from ascitic fluids. IHC images are from tumour tissue sections of associated tumour tissues. (For IF images, green – NuMA, red – α-tubulin, blue – DAPI in all panels) C. Summary of the mitotic defects observed in the NuMA stained tumour tissues. D. Summary of mitotic defects observed in the associated H&E stains.
(TIFF)

Figure S3 **Mitotic activity in cell cultures of malignant cells derived from ascitic fluids is mirrored in associated tumour tissue samples.** A) Tumour tissue sample 1101 with low mitotic activity (mitotic index in culture  =  0%). B) Tissue sample 1124 with high mitotic activity (mitotic index in culture  =  1.9%). Arrows indicate mitotic cells.
(PDF)

Figure S4 **Survival graph correlating NuMA expression and ovarian cancer patients’ survival data.** Preliminary data suggests that survival is associated with low NuMA levels in ovarian cancer patients. A moderate or strong NuMA score results in a three fold increase in the likelihood of death compared to a weak NuMA score (HR = 2.94, 95% CI [0.62, 13.95], LRT *p*-value  =  0.137). However, this result is not statistically significant.
(TIFF)
