# Supplementary figures and images for: NuMA Overexpression in Epithelial Ovarian Cancer
Source: PLoS One. 2012 Jun 14;7(6):e38945. doi: 10.1371/journal.pone.0038945 (PMC3375276; doi:10.1371/journal.pone.0038945)

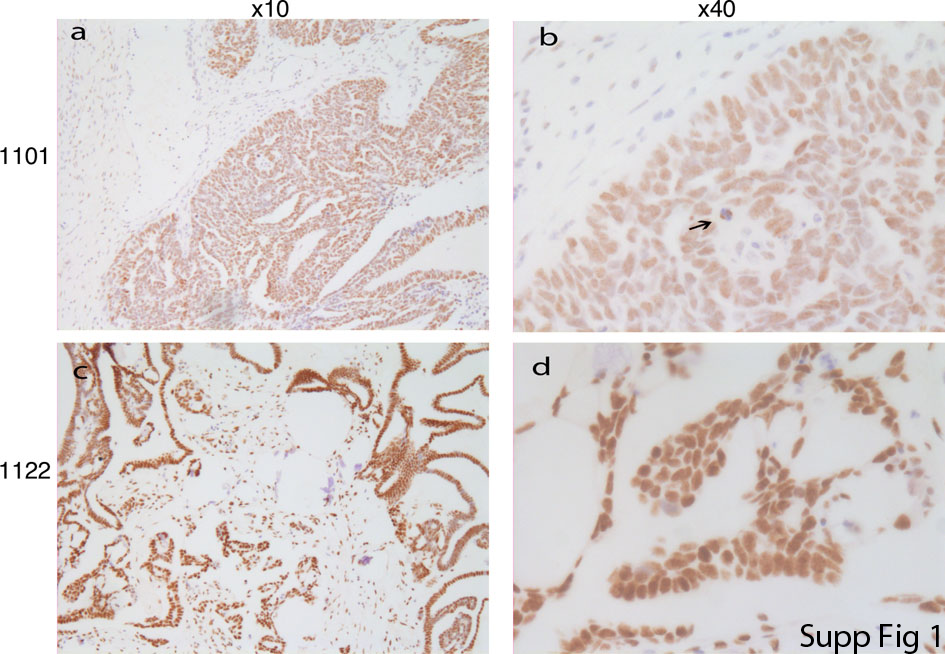

Supplement: Figure S1 — NuMA staining patterns in primary tumours correspond to staining intensities observed in primary cultures. Sections of primary tumour were immunostained for NuMA. (a,b) Weak nuclear staining was demonstrated in sample 1101; note spindle pole staining in mitotic cell (arrow) (b). (c,d) The strongest staining was observed in sample 1125. These findings mirror the results obtained by immunofluorescence analysis of the primary ascitic cultures derived from these tumours (Figure 4B). (TIFF) [file pone.0038945.s001.tiff]

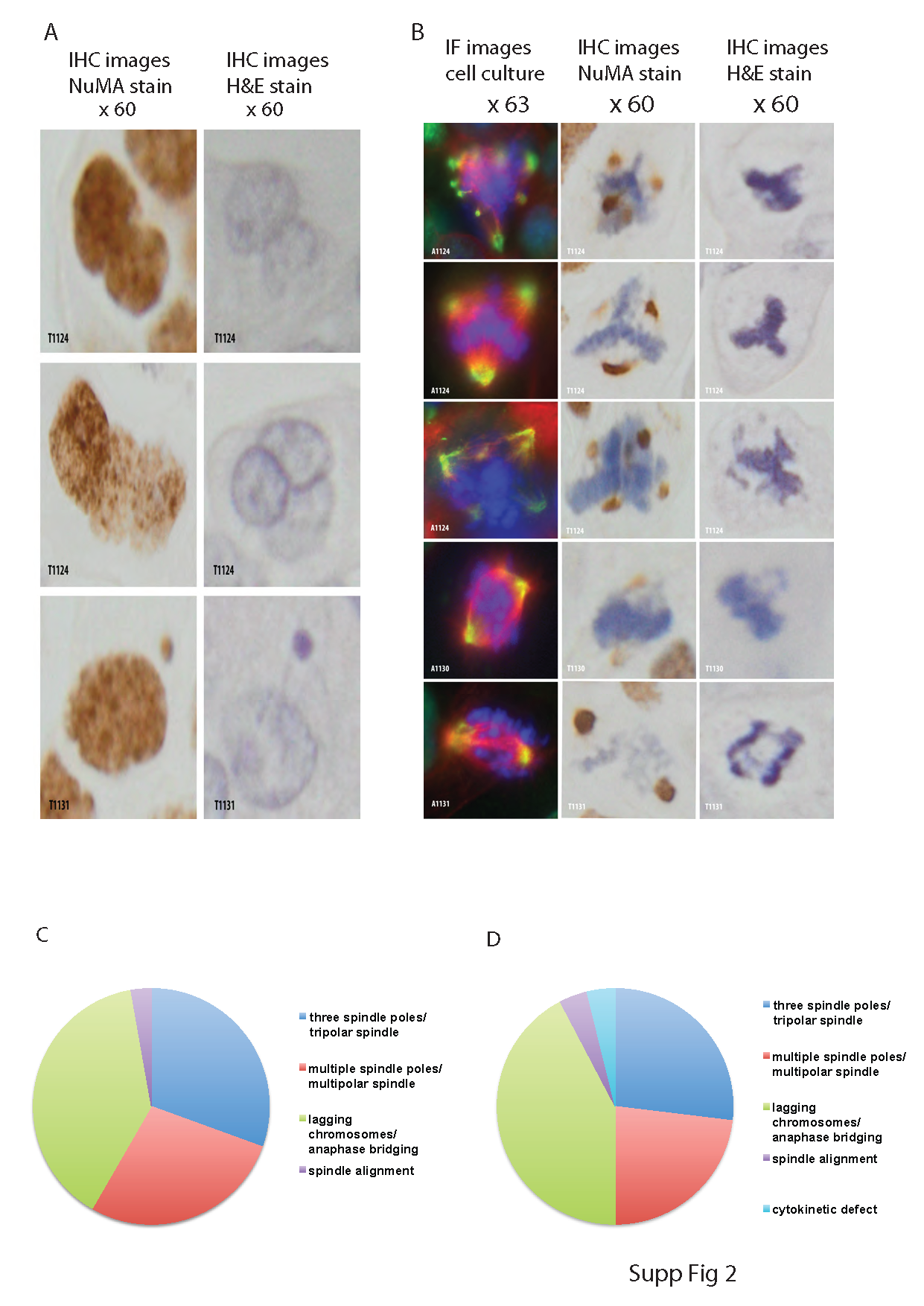

Supplement: Figure S2 — Interphase and mitotic defects in cell cultures of malignant cells derived from ascitic fluids are also present in associated tumour tissues. A. Binucleated and multinucleated cells and micronuclei are present in tumour tissues associated with ascitic cultures that also displayed these defects, as indicated by the NuMA and H&E staining. Number indicates patient ID, T indicates tumour sample. B. Various mitotic defects including multiple spindle poles, tripolar spindles, multipolar spindles and anaphase bridging are observed in cell cultures of malignant cells derived from ascitic fluids and in their associated tumours. IF images are from cell cultures of malignant cells derived from ascitic fluids. IHC images are from tumour tissue sections of associated tumour tissues. (For IF images, green – NuMA, red – α-tubulin, blue – DAPI in all panels) C. Summary of the mitotic defects observed in the NuMA stained tumour tissues. D. Summary of mitotic defects observed in the associated H&E stains. (TIFF) [file pone.0038945.s002.tiff]

A

IHC NuMA stain

IHC H&E stain

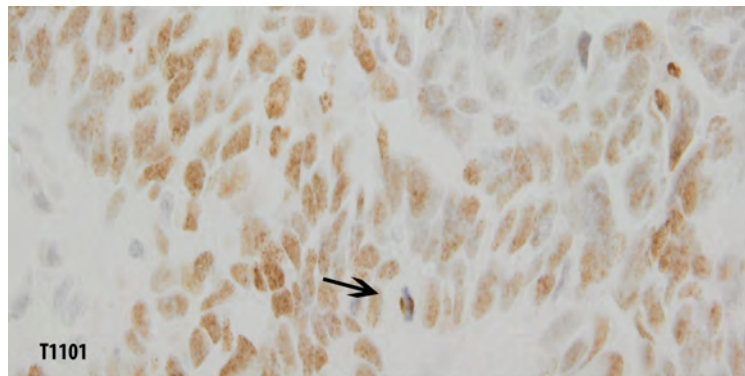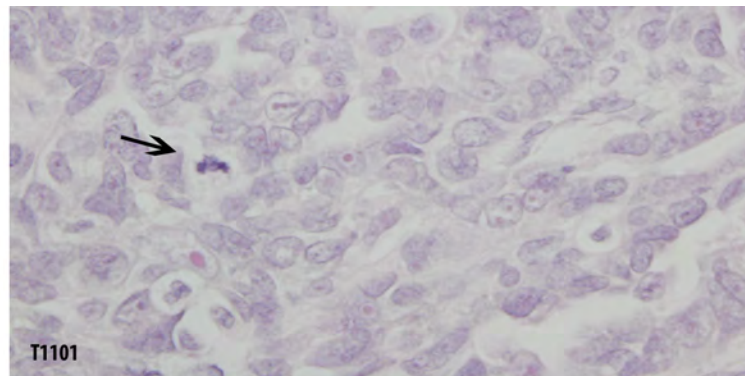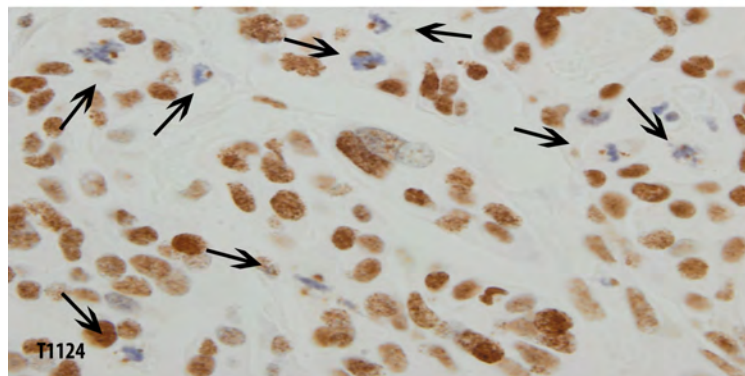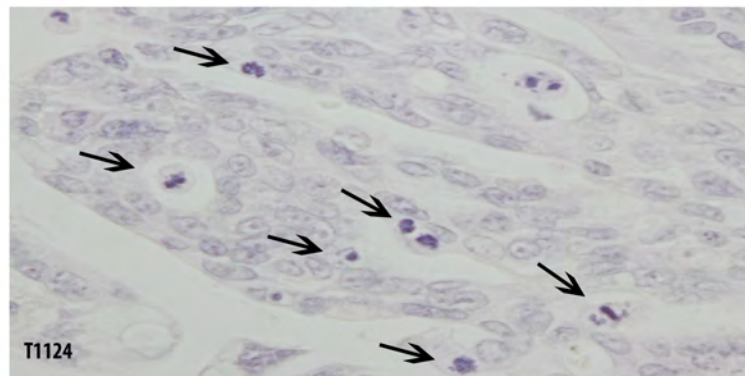

Supp Fig 3

Supplement: Figure S3 — Mitotic activity in cell cultures of malignant cells derived from ascitic fluids is mirrored in associated tumour tissue samples. A) Tumour tissue sample 1101 with low mitotic activity (mitotic index in culture = 0%). B) Tissue sample 1124 with high mitotic activity (mitotic index in culture = 1.9%). Arrows indicate mitotic cells. (PDF) [file pone.0038945.s003.pdf]

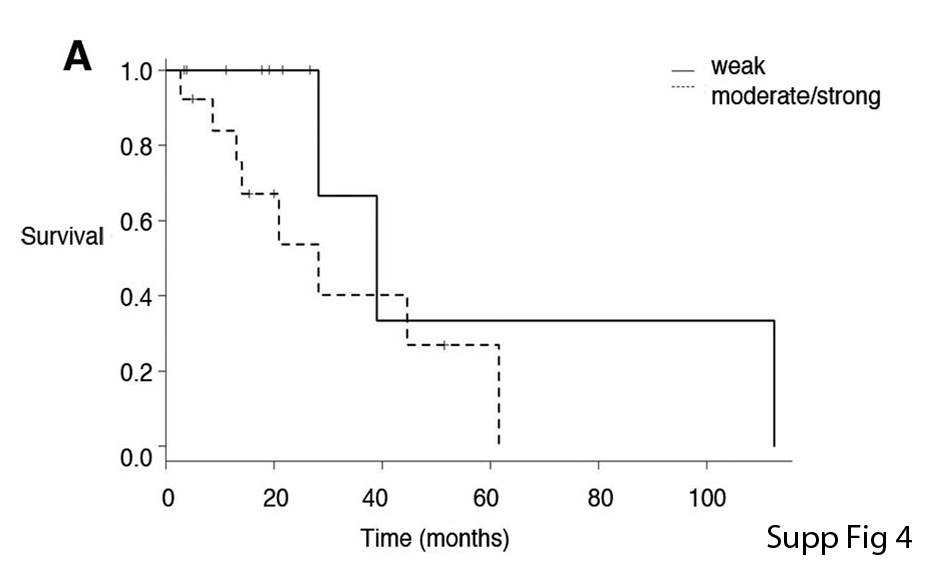

Supplement: Figure S4 — Survival graph correlating NuMA expression and ovarian cancer patients’ survival data. Preliminary data suggests that survival is associated with low NuMA levels in ovarian cancer patients. A moderate or strong NuMA score results in a three fold increase in the likelihood of death compared to a weak NuMA score (HR = 2.94, 95% CI [0.62, 13.95], LRT p-value = 0.137). However, this result is not statistically significant. (TIFF) [file pone.0038945.s004.tiff]
